# Supplementary material for: Modeling glioblastoma heterogeneity as a dynamic network of cell states
Source: Mol Syst Biol. 2021 Sep 16;17(9):e10105. doi: 10.15252/msb.202010105 (PMC8444284; doi:10.15252/msb.202010105)
Supplement: Supplementary file 6 — Source Data for Figure 5 [file MSB-17-e10105-s004.zip › Figure5A_sourcedata/GSEA_3017/nosigdb_stateA.GseaPreranked.1622795026485/RICHARDS_NOWAKOWSKI_SCIENCE_2017_MGE_IPC2_UPREG.html]

Details for gene set RICHARDS\_NOWAKOWSKI\_SCIENCE\_2017\_MGE\_IPC2\_UPREG[GSEA]

|  || Dataset | state53017 |
| Phenotype | NoPhenotypeAvailable |
| Upregulated in class | na\_pos |
| GeneSet | RICHARDS\_NOWAKOWSKI\_SCIENCE\_2017\_MGE\_IPC2\_UPREG |
| Enrichment Score (ES) | 0.34081843 |
| Normalized Enrichment Score (NES) | 2.8874154 |
| Nominal p-value | 0.0 |
| FDR q-value | 0.0 |
| FWER p-Value | 0.0 |
Table: GSEA Results Summary

  

Fig 1: Enrichment plot: RICHARDS\_NOWAKOWSKI\_SCIENCE\_2017\_MGE\_IPC2\_UPREG      
 Profile of the Running ES Score & Positions of GeneSet Members on the Rank Ordered List

  

| PROBE | GENE SYMBOL | GENE\_TITLE | RANK IN GENE LIST | RANK METRIC SCORE | RUNNING ES | CORE ENRICHMENT || 1 | CDK1 |  |  | 3 | 1.006 | 0.0123 | Yes |
| 2 | MKI67 |  |  | 4 | 0.926 | 0.0269 | Yes |
| 3 | KIF11 |  |  | 6 | 0.870 | 0.0395 | Yes |
| 4 | UBE2C |  |  | 7 | 0.793 | 0.0520 | Yes |
| 5 | MCM10 |  |  | 9 | 0.752 | 0.0627 | Yes |
| 6 | KIF20B |  |  | 10 | 0.749 | 0.0746 | Yes |
| 7 | BUB3 |  |  | 12 | 0.740 | 0.0851 | Yes |
| 8 | KPNA2 |  |  | 20 | 0.667 | 0.0872 | Yes |
| 9 | HIST1H4C |  |  | 21 | 0.659 | 0.0976 | Yes |
| 10 | ASPM |  |  | 27 | 0.639 | 0.1017 | Yes |
| 11 | CCNB1 |  |  | 37 | 0.599 | 0.1004 | Yes |
| 12 | CKS2 |  |  | 40 | 0.587 | 0.1072 | Yes |
| 13 | NCAPG |  |  | 44 | 0.556 | 0.1124 | Yes |
| 14 | HJURP |  |  | 47 | 0.539 | 0.1186 | Yes |
| 15 | CKS1B |  |  | 50 | 0.530 | 0.1245 | Yes |
| 16 | DBF4 |  |  | 55 | 0.514 | 0.1279 | Yes |
| 17 | SMC3 |  |  | 58 | 0.504 | 0.1334 | Yes |
| 18 | KIF14 |  |  | 59 | 0.503 | 0.1414 | Yes |
| 19 | MASTL |  |  | 60 | 0.499 | 0.1493 | Yes |
| 20 | CENPE |  |  | 62 | 0.496 | 0.1559 | Yes |
| 21 | MIS18BP1 |  |  | 64 | 0.495 | 0.1625 | Yes |
| 22 | CCNA2 |  |  | 65 | 0.495 | 0.1704 | Yes |
| 23 | TPX2 |  |  | 66 | 0.488 | 0.1781 | Yes |
| 24 | MYBL2 |  |  | 69 | 0.486 | 0.1833 | Yes |
| 25 | DLGAP5 |  |  | 76 | 0.475 | 0.1836 | Yes |
| 26 | HIST1H1B |  |  | 77 | 0.475 | 0.1912 | Yes |
| 27 | CDC20 |  |  | 80 | 0.472 | 0.1962 | Yes |
| 28 | VRK1 |  |  | 82 | 0.470 | 0.2024 | Yes |
| 29 | UBE2T |  |  | 83 | 0.470 | 0.2099 | Yes |
| 30 | DUT |  |  | 87 | 0.466 | 0.2136 | Yes |
| 31 | TUBB4B |  |  | 93 | 0.462 | 0.2149 | Yes |
| 32 | CENPF |  |  | 94 | 0.461 | 0.2222 | Yes |
| 33 | TOP2A |  |  | 95 | 0.457 | 0.2294 | Yes |
| 34 | HIST1H1D |  |  | 98 | 0.456 | 0.2343 | Yes |
| 35 | ZWINT |  |  | 106 | 0.447 | 0.2329 | Yes |
| 36 | AURKB |  |  | 108 | 0.446 | 0.2387 | Yes |
| 37 | CDCA8 |  |  | 115 | 0.437 | 0.2384 | Yes |
| 38 | KIF23 |  |  | 116 | 0.436 | 0.2453 | Yes |
| 39 | DEPDC1 |  |  | 119 | 0.432 | 0.2498 | Yes |
| 40 | MELK |  |  | 123 | 0.427 | 0.2529 | Yes |
| 41 | BUB1B |  |  | 129 | 0.421 | 0.2535 | Yes |
| 42 | HELLS |  |  | 135 | 0.417 | 0.2541 | Yes |
| 43 | RTKN2 |  |  | 141 | 0.413 | 0.2546 | Yes |
| 44 | GTSE1 |  |  | 146 | 0.411 | 0.2563 | Yes |
| 45 | CCNB2 |  |  | 147 | 0.411 | 0.2628 | Yes |
| 46 | ANLN |  |  | 152 | 0.407 | 0.2645 | Yes |
| 47 | MAD2L1 |  |  | 153 | 0.406 | 0.2709 | Yes |
| 48 | LINC01224 |  |  | 154 | 0.406 | 0.2773 | Yes |
| 49 | PBX3 |  |  | 157 | 0.404 | 0.2813 | Yes |
| 50 | KIFC1 |  |  | 160 | 0.402 | 0.2852 | Yes |
| 51 | ARHGAP11A |  |  | 162 | 0.399 | 0.2903 | Yes |
| 52 | KIF2C |  |  | 165 | 0.397 | 0.2942 | Yes |
| 53 | CCAR1 |  |  | 170 | 0.395 | 0.2956 | Yes |
| 54 | MCM7 |  |  | 171 | 0.394 | 0.3019 | Yes |
| 55 | NDC80 |  |  | 173 | 0.393 | 0.3069 | Yes |
| 56 | BUB1 |  |  | 188 | 0.384 | 0.2961 | Yes |
| 57 | DCLRE1C |  |  | 190 | 0.381 | 0.3010 | Yes |
| 58 | KIF22 |  |  | 197 | 0.377 | 0.2997 | Yes |
| 59 | PARD3 |  |  | 200 | 0.375 | 0.3032 | Yes |
| 60 | CDCA3 |  |  | 203 | 0.372 | 0.3067 | Yes |
| 61 | PTMA |  |  | 206 | 0.371 | 0.3102 | Yes |
| 62 | SMC4 |  |  | 217 | 0.367 | 0.3039 | Yes |
| 63 | SPC25 |  |  | 226 | 0.363 | 0.3001 | Yes |
| 64 | NCAPH |  |  | 232 | 0.359 | 0.2997 | Yes |
| 65 | SOX6 |  |  | 234 | 0.358 | 0.3042 | Yes |
| 66 | ZNF704 |  |  | 235 | 0.357 | 0.3098 | Yes |
| 67 | H2AFZ |  |  | 245 | 0.351 | 0.3046 | Yes |
| 68 | FEN1 |  |  | 259 | 0.343 | 0.2943 | Yes |
| 69 | TTK |  |  | 262 | 0.340 | 0.2973 | Yes |
| 70 | TUBB |  |  | 264 | 0.340 | 0.3015 | Yes |
| 71 | CENPJ |  |  | 270 | 0.339 | 0.3008 | Yes |
| 72 | CLSPN |  |  | 274 | 0.336 | 0.3025 | Yes |
| 73 | DEPDC1B |  |  | 275 | 0.335 | 0.3078 | Yes |
| 74 | ESCO2 |  |  | 282 | 0.333 | 0.3059 | Yes |
| 75 | ORC6 |  |  | 283 | 0.333 | 0.3111 | Yes |
| 76 | C21orf58 |  |  | 294 | 0.328 | 0.3043 | Yes |
| 77 | G2E3 |  |  | 302 | 0.325 | 0.3010 | Yes |
| 78 | MND1 |  |  | 304 | 0.324 | 0.3049 | Yes |
| 79 | RAD18 |  |  | 307 | 0.324 | 0.3076 | Yes |
| 80 | BRCA2 |  |  | 308 | 0.323 | 0.3128 | Yes |
| 81 | CENPN |  |  | 310 | 0.323 | 0.3167 | Yes |
| 82 | MLLT10 |  |  | 311 | 0.322 | 0.3218 | Yes |
| 83 | HNRNPDL |  |  | 315 | 0.320 | 0.3232 | Yes |
| 84 | FANCI |  |  | 319 | 0.319 | 0.3247 | Yes |
| 85 | DNA2 |  |  | 321 | 0.318 | 0.3285 | Yes |
| 86 | RFC3 |  |  | 337 | 0.314 | 0.3154 | Yes |
| 87 | CDCA5 |  |  | 341 | 0.312 | 0.3167 | Yes |
| 88 | CLIC1 |  |  | 342 | 0.311 | 0.3216 | Yes |
| 89 | CHD7 |  |  | 343 | 0.311 | 0.3266 | Yes |
| 90 | PRC1 |  |  | 350 | 0.310 | 0.3242 | Yes |
| 91 | ANP32B |  |  | 357 | 0.307 | 0.3219 | Yes |
| 92 | ECT2 |  |  | 365 | 0.305 | 0.3183 | Yes |
| 93 | CCNF |  |  | 366 | 0.305 | 0.3231 | Yes |
| 94 | TTF2 |  |  | 367 | 0.305 | 0.3279 | Yes |
| 95 | CDCA2 |  |  | 369 | 0.304 | 0.3315 | Yes |
| 96 | BRCA1 |  |  | 373 | 0.302 | 0.3327 | Yes |
| 97 | CKAP2 |  |  | 379 | 0.301 | 0.3314 | Yes |
| 98 | RRM2 |  |  | 383 | 0.300 | 0.3326 | Yes |
| 99 | XRCC2 |  |  | 384 | 0.300 | 0.3373 | Yes |
| 100 | CENPW |  |  | 386 | 0.299 | 0.3408 | Yes |
| 101 | RACGAP1 |  |  | 399 | 0.293 | 0.3310 | No |
| 102 | HMGB1 |  |  | 403 | 0.292 | 0.3320 | No |
| 103 | GAD1 |  |  | 423 | 0.288 | 0.3137 | No |
| 104 | HMGB2 |  |  | 424 | 0.288 | 0.3183 | No |
| 105 | PID1 |  |  | 429 | 0.287 | 0.3180 | No |
| 106 | NUSAP1 |  |  | 430 | 0.286 | 0.3225 | No |
| 107 | NUF2 |  |  | 434 | 0.285 | 0.3234 | No |
| 108 | MIS18A |  |  | 439 | 0.284 | 0.3231 | No |
| 109 | NCAPD2 |  |  | 440 | 0.283 | 0.3276 | No |
| 110 | LMNB1 |  |  | 442 | 0.282 | 0.3308 | No |
| 111 | CDC25C |  |  | 443 | 0.282 | 0.3353 | No |
| 112 | KIF20A |  |  | 447 | 0.281 | 0.3361 | No |
| 113 | ANP32E |  |  | 453 | 0.280 | 0.3345 | No |
| 114 | ATAD2 |  |  | 470 | 0.275 | 0.3196 | No |
| 115 | EZH2 |  |  | 471 | 0.275 | 0.3240 | No |
| 116 | SKA1 |  |  | 475 | 0.274 | 0.3247 | No |
| 117 | EFS |  |  | 487 | 0.272 | 0.3158 | No |
| 118 | BLM |  |  | 490 | 0.272 | 0.3177 | No |
| 119 | KIF4A |  |  | 504 | 0.269 | 0.3063 | No |
| 120 | HAUS8 |  |  | 518 | 0.267 | 0.2949 | No |
| 121 | ZNF90 |  |  | 520 | 0.266 | 0.2979 | No |
| 122 | RCC1 |  |  | 528 | 0.265 | 0.2936 | No |
| 123 | SKA3 |  |  | 531 | 0.264 | 0.2954 | No |
| 124 | ALYREF |  |  | 535 | 0.264 | 0.2960 | No |
| 125 | KIF15 |  |  | 538 | 0.263 | 0.2977 | No |
| 126 | CDKN3 |  |  | 539 | 0.263 | 0.3019 | No |
| 127 | TTF1 |  |  | 541 | 0.262 | 0.3048 | No |
| 128 | STIL |  |  | 545 | 0.261 | 0.3054 | No |
| 129 | CENPO |  |  | 549 | 0.260 | 0.3058 | No |
| 130 | FBXO5 |  |  | 557 | 0.258 | 0.3015 | No |
| 131 | DSEL |  |  | 559 | 0.258 | 0.3044 | No |
| 132 | DDX39A |  |  | 562 | 0.258 | 0.3061 | No |
| 133 | TMPO |  |  | 584 | 0.253 | 0.2848 | No |
| 134 | HIST1H2BH |  |  | 595 | 0.251 | 0.2767 | No |
| 135 | ABAT |  |  | 617 | -0.259 | 0.2555 | No |
| 136 | FAM111A |  |  | 618 | -0.259 | 0.2596 | No |
| 137 | ARHGEF26 |  |  | 628 | -0.262 | 0.2529 | No |
| 138 | MAGI1 |  |  | 642 | -0.266 | 0.2415 | No |
| 139 | RAB3IP |  |  | 667 | -0.280 | 0.2171 | No |
| 140 | SMARCA2 |  |  | 711 | -0.310 | 0.1702 | No |
| 141 | ZEB2 |  |  | 722 | -0.319 | 0.1632 | No |
| 142 | GRIK3 |  |  | 747 | -0.341 | 0.1397 | No |
| 143 | NEDD4L |  |  | 764 | -0.355 | 0.1261 | No |
| 144 | DLX1 |  |  | 770 | -0.361 | 0.1258 | No |
| 145 | MSI2 |  |  | 771 | -0.361 | 0.1315 | No |
| 146 | EPHA4 |  |  | 778 | -0.367 | 0.1301 | No |
| 147 | DST |  |  | 818 | -0.400 | 0.0895 | No |
| 148 | ST8SIA5 |  |  | 849 | -0.443 | 0.0604 | No |
| 149 | TMSB4X |  |  | 850 | -0.443 | 0.0674 | No |
| 150 | DCLK2 |  |  | 859 | -0.449 | 0.0649 | No |
| 151 | RGS12 |  |  | 880 | -0.504 | 0.0488 | No |
| 152 | GPC6 |  |  | 896 | -0.540 | 0.0393 | No |
| 153 | KCNJ2 |  |  | 921 | -0.618 | 0.0202 | No |
| 154 | CDH2 |  |  | 947 | -0.762 | 0.0021 | No |
| 155 | RND3 |  |  | 959 | -0.864 | 0.0026 | No |
| 156 | ZBTB20 |  |  | 964 | -0.895 | 0.0119 | No |
| 157 | CDK6 |  |  | 966 | -0.920 | 0.0253 | No |
Table: GSEA details [plain text format]

  

Fig 2: RICHARDS\_NOWAKOWSKI\_SCIENCE\_2017\_MGE\_IPC2\_UPREG: Random ES distribution      
 Gene set null distribution of ES for **RICHARDS\_NOWAKOWSKI\_SCIENCE\_2017\_MGE\_IPC2\_UPREG**
